# Reduced Variability of Ongoing and Evoked Cortical Activity Leads to Improved Behavioral Performance

**DOI:** 10.1371/journal.pone.0043166

**Published:** 2012-08-24

**Authors:** Anders Ledberg, Anna Montagnini, Richard Coppola, Steven L. Bressler

**Affiliations:** 1 Center of Brain and Cognition, Department of Information and Communication Technologies, Universitat Pompeu Fabra, Barcelona, Spain; 2 Institut de Neurosciences de la Timone, Centre National de la Recherche Scientifique and Aix-Marseille University, Marseille, France; 3 Clinical Brain Disorders Branch, National Institute of Mental Health, Bethesda, Maryland, United States of America; 4 Center for Complex Systems and Brain Sciences, Department of Psychology, Florida Atlantic University, Boca Raton, Florida, United States of America; Indiana University, United States of America

## Abstract

Sensory responses of the brain are known to be highly variable, but the origin and functional relevance of this variability have long remained enigmatic. Using the variable foreperiod of a visual discrimination task to assess variability in the primate cerebral cortex, we report that visual evoked response variability is not only tied to variability in ongoing cortical activity, but also predicts mean response time. We used cortical local field potentials, simultaneously recorded from widespread cortical areas, to gauge both ongoing and visually evoked activity. Trial-to-trial variability of sensory evoked responses was strongly modulated by foreperiod duration and correlated both with the cortical variability before stimulus onset as well as with response times. In a separate set of experiments we probed the relation between small saccadic eye movements, foreperiod duration and manual response times. The rate of eye movements was modulated by foreperiod duration and eye position variability was positively correlated with response times. Our results indicate that when the time of a sensory stimulus is predictable, reduction in cortical variability before the stimulus can improve normal behavioral function that depends on the stimulus.

## Introduction

In sensory areas of the brain, neuronal responses to the same stimulus may vary considerably from one trial to the next [1]–[4] and this variability is weakly correlated between neighboring neurons [5], [6]. Since neuronal trial-to-trial variability could potentially interfere with the organism's ability to utilize sensory information to guide behavior, it is essential to know whether it is reduced in normal behavior and how this reduction comes about.

Recent studies show that neuronal trial-to-trial variability and covariability in widespread cortical areas are reduced by the onset of a sensory stimulus [7] and that behaviorally relevant stimuli may cause further variability reduction [8]–[10]. The stimulus-induced reduction in variability is partly independent of the state of the subjects since the effect can be observed both in anesthetized and awake animals [7]. On the other hand, others have interpreted the variability reduction as a correlate of visual selective attention [8]–[10].

Neuronal response variability may have several causes, and in the case of visual stimuli, subtle between trial differences in eye movements are a prominent such cause [11]. Since both macro- and micro saccades influence the firing of single neurons [12]–[14] as well as the amplitude of local field potentials [15], [16] one possible way to reduce trial-to-trial variability of the cortical response is to reduce the rate of saccadic eye movements. Recent evidence obtained in both human- [17] and nonhuman primates [18], indicates that microsaccades in close temporal relation to the imperative stimulus lead to impaired behavioral performance, an observation suggesting that cortical response variability, induced by eye movements, interferes with normal behavior.

How can neuronal activity induced by, or at least time-locked to, saccadic eye movements affect the variability of the cortical response to a sensory stimulus? Sensory evoked cortical responses depend on the state of ongoing cortical activity at the time of stimulus presentation [19]–[21]. The brain may therefore reduce sensory response variability by reducing the variability of ongoing cortical activity. Part of the cortical (co-)variability reduction previously reported in visual area V4 has in fact been attributed to a reduction of background cortical activity [9]. A long-standing hypothesis in systems neuroscience holds that cortical state is controlled by ascending neuromodulator systems [22], [23] and it has recently been shown that activating ascending systems improves the coding of sensory stimuli, partly through a reduction in trial-to-trial variability [24]. When the time-point of the imperative stimulus is to some extent predictable it is therefore possible that the organism reduces cortical variability by decreasing the rate of saccadic eye movements or by activating an ascending system, or both. Likely these mechanisms are not independent.

Here we investigate the hypothesis that reduced variability in ongoing cortical activity leads to reduced variability in the sensory evoked response, which in turn leads to improved behavioral performance. We furthermore provide evidence that suggests that the reduced variability is partly but not completely a consequence of a reduction in the rate of small eye movements. A visual discrimination task was used in which subjects had to make a swift motor response to a briefly presented visual stimulus. The task design allowed cortical activity and mean response time to be evaluated in relation to foreperiod (time between trial onset and stimulus onset) [25]–[27]. Cortical activity was assessed by local field potentials (LFPs), which reflect local synaptic activity [28], [29] and thus provide a sensitive measure of ongoing cortical activity. The strong correlation known to exist between the initial LFP response to a visual stimulus (visual evoked potential; VEP) and the corresponding multi-unit spiking response [30], [31] implies that VEP variability is related to the (co-)variability of single-neuron activity. We show that VEP variability in widespread cortical regions is strongly modulated by foreperiod duration, that VEP variability depends on ongoing cortical activity at the time of stimulus onset, and that foreperiod-dependent reduction of mean response times can be explained by the reduction in VEP variability. Small eye movement were not monitored in the LFP experiments so we adopted the same behavioral task for human subjects and run an experiment in which eye movements were measured with high spatio-temporal precision. We show that the rate of small saccades decreases as a function of foreperiod duration and that the rate of saccades is correlated with reaction time. Taken together our results support a model in which subjects actively control neuronal variability, partly by reducing eye movements, in order to optimize performance.

## Results

**Figure 1:**
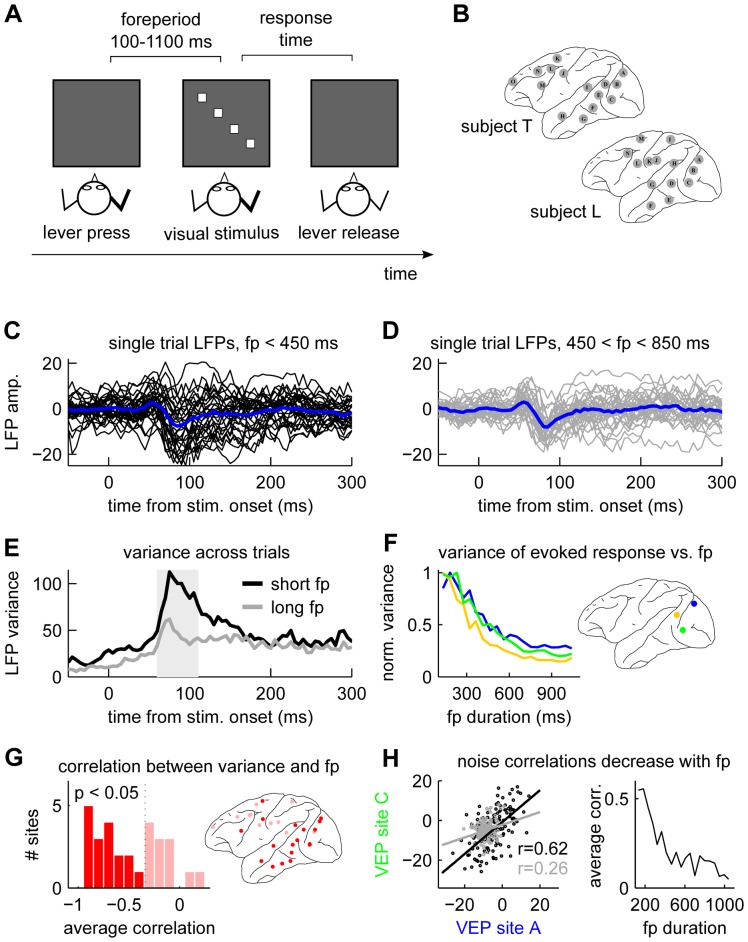
Foreperiod duration modulates trial-to-trial variability of the evoked response. (**A**) Outline of main components of the behavioral task. (**B**) Brain maps showing the approximate location of the recording sites for the two subjects. (**C**) 30 single-trial LFPs from one recording site (E in subject T) with foreperiod durations 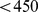 ms, LFP units arbitrary. (**D**) same as **C** but with foreperiods between 450 and 850 ms. (**E**) Sample variance (over trials) of the LFPs for the data shown in **C** and **D**. (**F**) LFP variability as a function of foreperiod duration for three example sites from subject T. The mean sample variance in a time-window indicated by the shaded box in **E** was calculated in 20 groups of trials sorted by foreperiod duration. The values are normalized to a maximum value of 1 for comparison between sites. Colored disks indicate the locations of the sites. (**G**) Rank correlations between VEP variability and foreperiod durations for all sites. Sites with significant correlations (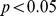 corrected for multiple comparisons) are shown in dark red both in the histogram and on the cortical map. (**H**) Noise correlations decrease with increasing foreperiod duration. Left panel shows how the VEP at one site (green color in panel **F**) depends on that of another site (blue color in panel **F**) for two groups of trials with different foreperiod duration (black dots: foreperiods 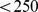 ms; gray dots foreperiods 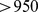). Right panel shows how the noise correlations decrease as a function of foreperiod duration (calculations based on the same 20 groups of trials used in **F** and **G**).

To characterize the variability of stimulus evoked cortical activity, we analyzed LFPs simultaneously recorded from multiple cortical areas in two monkeys performing a visual discrimination task (Figure 1A,B and Material & Methods). A critical aspect of the task design was that the foreperiod varied randomly from trial to trial (rectangular distribution with 60 equally sized bins between 100 and 1100 ms).

### VEP variability is modulated by foreperiod duration

Previously we showed that LFPs recorded from widespread cortical areas have a short-latency (50–100 ms) VEP response, time-locked to the onset of the visual stimulus [32]. Figure 1 shows that early VEP amplitude variability strongly depended on foreperiod duration: single-trial LFPs from primary visual cortex had larger trial-to-trial variability after short (Figure 1C) than long (Figure 1D) foreperiods. The variance across trials, computed at each time point, was substantially lower after long than short foreperiods (Figure 1E). To investigate the generality of this finding, we divided the trials into 20 non-overlapping groups based on foreperiod duration. In each group we computed the variance over trials in a 50 ms time window covering the early VEP (the window is indicated by the gray rectangle in Figure 1E). The average within-window variability decreased markedly as foreperiod duration increased, as illustrated by three example sites in Figure 1F. At a large number of sites in the two monkeys, trial-to-trial variability of the evoked response significantly declined with increasing foreperiod duration, as measured by rank correlation (Figure 1G). The effect was strongest in occipital and temporal regions (‘visual areas’) but was also observed at frontal sites.

We next tested whether *co-variability* in the evoked response between different sites (so-called noise correlations [33]) was also modulated by foreperiod duration. The left panel in Figure 1H shows a scatter plot of the average early VEP amplitude at two sites in primary visual cortex for groups of trials with short (black) and long (gray) foreperiod durations. The linear relationship between the early VEP amplitudes at these two sites is much stronger for short foreperiods, indicating that noise correlation decreases with increasing foreperiod duration. The right panel in Figure 1H shows that the correlation decreases monotonically as a function of foreperiod duration. Of all the 73 site pairs that had a significant decrease in variance at both sites, six had a significant decline in noise correlation as a function of foreperiod duration.

### Ongoing cortical activity is modulated by foreperiod duration

**Figure 2:**
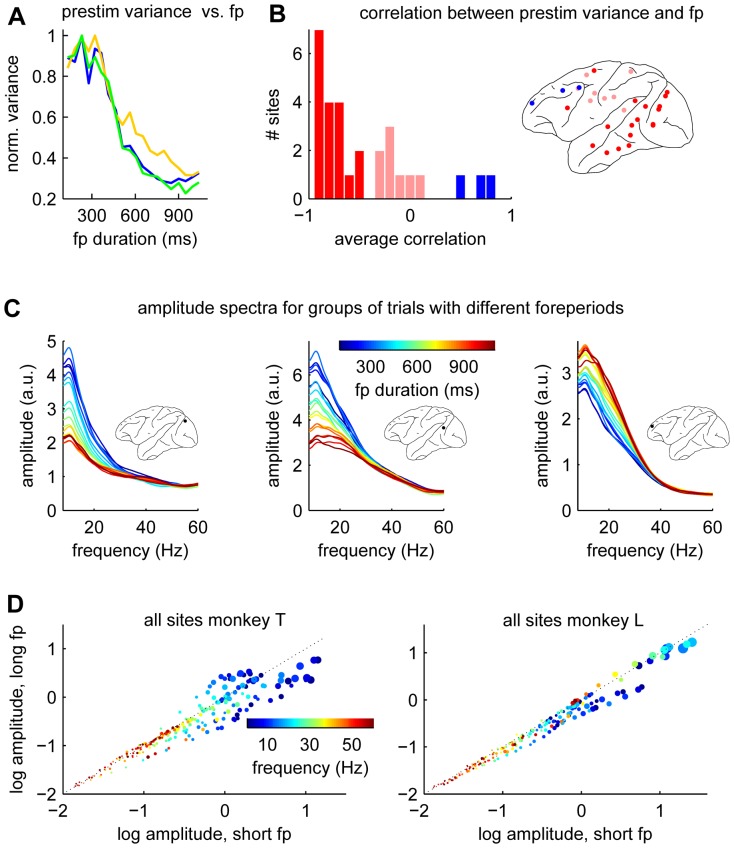
Variability of prestimulus activity depends on foreperiod duration. (**A**) Normalized variance of the prestimulus data as a function of foreperiod duration for the same three sites shown in Figure 1F. Same color code as in that figure. (**B**) Histogram showing correlation coefficients between prestimulus variance and foreperiod duration. Data from both monkeys. Sites with a significant (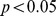 corrected for multiple comparisons) negative correlation in dark red. Sites with a significant positive correlation in blue. Pink color signifies non-significant cases. The brain map to the right shows the corresponding locations. (**C**) Amplitude spectra as a function of foreperiod duration for three representative sites from monkey T. The different foreperiod groups are indicated by the color of the spectra according to the color bar in the mid-panel. The locations of the sites are shown by the three brain maps. (**D**) Scatter plots of the log average amplitudes for two groups of foreperiods (short 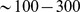 ms; long 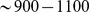 ms). Each point of a particular color corresponds to a recording site. The different colors corresponds to frequencies according to the color bar shown. The area of a dot is proportional to the variability of the corresponding data. The black dotted line (the diagonal) shows the points where the amplitudes are the same for short and long foreperiods. That is, the further a point is from this line the larger is the dependence on foreperiod duration. In monkey T (to the left) three sites had amplitudes that increased as a function of foreperiod duration (see **B**). These sites corresponds to the points above the diagonal.

Is the reduction in VEP variability related to changes in the ongoing cortical activity? This is suggested by Figure 1C–E where it can be seen that the variability before stimulus onset also depends on foreperiod duration. To investigate this issue further we first analyzed the LFPs in a 100 ms time window extending from 90 ms before to 10 ms after the onset of the visual stimulus (the prestimulus epoch, before the stimulus evoked activity reached the cortical level) and found that trial-to-trial variability of ongoing cortical activity was strongly modulated by foreperiod duration at many sites (Figure 2A,B). Of all the sites with a significant change in VEP variability (as seen in Figure 1G) all except one also had a significant change in prestimulus trial-to-trial variability (compare Figure 1G with Figure 2B). Three sites from monkey T had prestimulus variability that increased with foreperiod duration (blue dots in Figure 2B). None of these sites had VEP variability that depended on foreperiod duration. A spectral analysis of the prestimulus epoch showed that the foreperiod duration dependent changes in variability occurred mainly at frequencies below 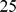 Hz (Figure 2C,D).

### Ongoing cortical activity predicts VEP variability

**Figure 3:**
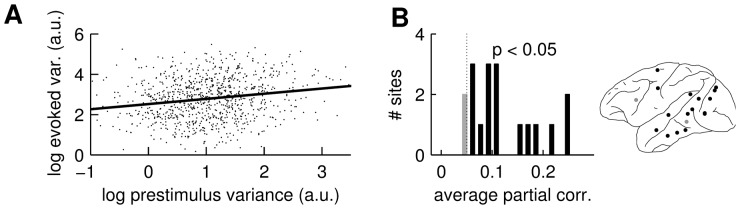
Variability in the evoked response is correlated with prestimulus variability. (**A**) Single-trial variability of the prestimulus LFP versus single-trial variability of the evoked response. The points correspond to single-trial data from one site (E) in subject T. The solid line shows the best linear fit. The linear correlation highly significant (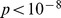). (**B**) The histogram shows the distribution of mean partial correlation coefficients between prestimulus and evoked variability for all the sites in the two subjects having significantly modulated evoked variability. The dotted line marks the level at which the mean partial correlation coefficients are significantly different from zero (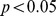 corrected for multiple comparisons). The cortical map shows the site locations in tones (black or gray) corresponding to those in the histogram.

Next we tested whether evoked response variability did in fact depend on the variability of ongoing cortical activity by comparing VEP and prestimulus variability on a trial-by-trial basis. Figure 3A shows that these variables can indeed be significantly correlated (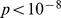, linear regression analysis), indicating that the degree of variability in the prestimulus period is indicative of the level of evoked response variability. To control for the possibility that changes in evoked (Figure 1) and prestimulus (Figure 2) variability were independently caused by foreperiod duration, we computed the correlation between the variabilities in prestimulus and evoked epochs with the effect of foreperiod ‘partialed out’. Figure 3B shows that at most sites these partial correlation coefficients were significantly greater than zero (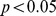, corrected for multiple comparisons). The correlations were non-significant at only two of the 17 sites where the VEP variability was modulated by foreperiod duration. This is a strong indication that VEP variability is causally related to the variability of the ongoing cortical activity.

### VEP variability predicts mean response times

**Figure 4:**
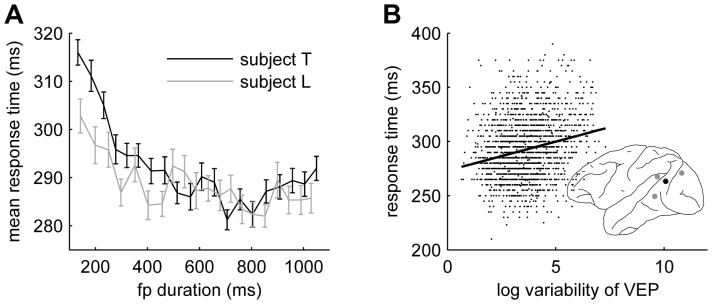
Response time is is correlated with foreperiod (fp) duration and VEP variability. (**A**) Mean response times for the two subjects as a function of fp duration (solid lines). Error bars show the standard errors. (**B**) Single-trial response times versus VEP variability estimate for one recording site in subject T. Solid line shows best linear fit. Inset show the sites that had a significant correlation between VEP variability and response time (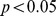, corrected for multiple comparisons). The black dot in the cortical map indicates the site corresponding to the scatter plot.

Mean response times decreased as a function of foreperiod duration (Figure 4A), replicating earlier studies [26], [27]. This decrease was not due to a speed-accuracy trade-off since the percent correct responses (average 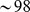
% and 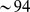
% for the two subjects) did not decline with increasing foreperiod duration (not shown). The mean response time decrease was highly significant (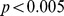 and 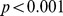 for the two subjects respectively), and the shape of the decrease resembled that of the variability modulation (cf. Figure 1F). To test for a relation between response time and cortical variability we examined the covariation of these two variables at the single-trial level. Single-trial response times tended to shorten as cortical variability at many sites declined (shown for an extrastriate recording site in Figure 3B). Since both cortical variability and response time were influenced by foreperiod duration we computed the partial correlation between those two variables, thus removing the variability induced by foreperiod duration. Of the 17 sites with significantly modulated variability, four had a significant partial correlation between cortical variability and response time (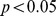, corrected for 17 multiple comparisons). The locations of these four sites are shown in the cortical map of Figure 4B.

### The role of eye movements

**Figure 5:**
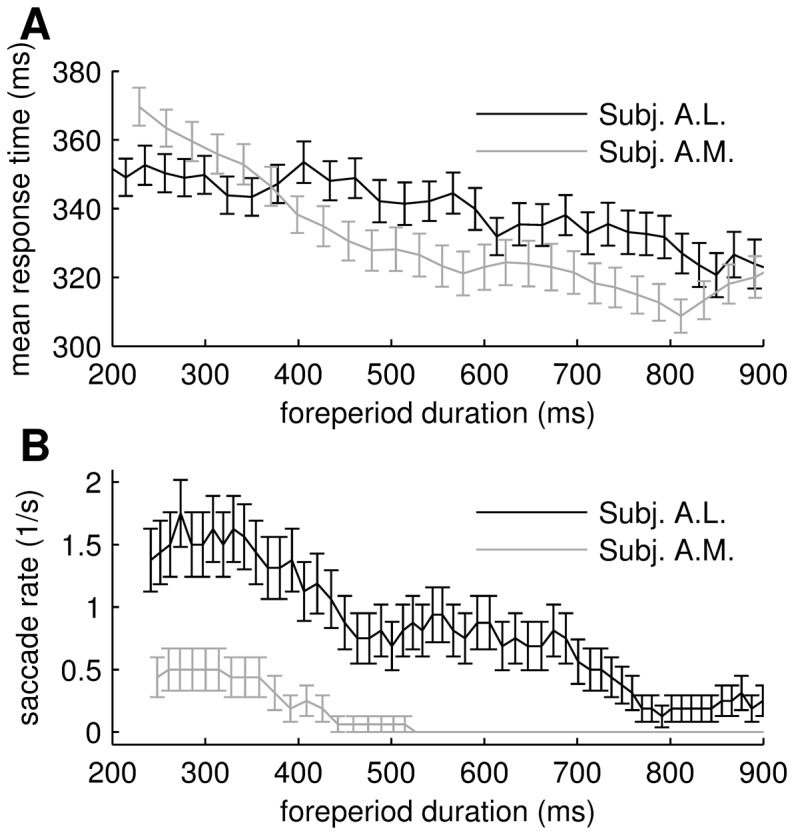
Rate of small eye movements is modulated by foreperiod duration. (**A**) Mean response times as a function of foreperiod duration. Error bars show standard errors. (**B**) Rate of small saccades in a 200 ms window immediately preceding stimulus onset. Error bars show standard errors.

Small eye movements were not monitored in the monkey experiments. However, the task is demanding and the stimulus was presented for 100 ms which is probably too short time for saccadic eye movements to be beneficial in solving the task. To further investigate the relation between eye movements and response behavior in our task we ran behavioral experiments in two human subjects using exactly the same task while monitoring eye movements. Figure 5A shows that the effect of foreperiod duration on mean response times is present also in the human subjects. The effect was statistically significant in both subjects (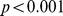, linear regression analysis). Next we investigated if the saccadic eye movements were influenced by the foreperiod duration. Since the saccade rate might be affected by the visual stimulus we analyzed the saccades made in a 200 ms window immediately preceding stimulus onset. We further restricted the analysis to trials where subjects were not moving their eyes more than two degrees of visual angle (these trials comprise more than 98 percent of all trials). Figure 5B shows that the rate of these small eye movements was strongly modulated by foreperiod duration. The effect was statistically significant in both subjects (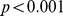). We next asked if eye movements alone could account for the reduction in response times. This was assessed by computing the correlation between foreperiod duration and response time, after eye movements had been partialed out (partial correlation analysis). This analysis showed that there was still a strong effect of foreperiod duration on response times in one subject (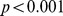) and a trend in the other (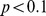). To substantiate these result further, we restricted the analysis to trials where the subjects did not make any noticeable saccadic eye movements within the 200 ms window immediately preceding stimulus onset. The effect of foreperiod on mean response time was still statistically significant.

These results demonstrate two things. First that the probability of making small eye movements decreases as a function of foreperiod duration; and second that the effect of foreperiod duration on mean response times is not only a consequence of these eye movements.

### How does increased VEP variability lead to increased mean RT?

We found a correlation between variability of sensory evoked responses and response times (Figure 4B) and in this section we describe a phenomenological model accounting for this finding. To link VEP variability to response times we use a standard model of sensory decision making (below). However, this model is not formulated at the level of neural data and we therefore first need to consider how to connect VEP variability to variables that appear in the model. We use information theory to establish this connection.

**Figure 6:**
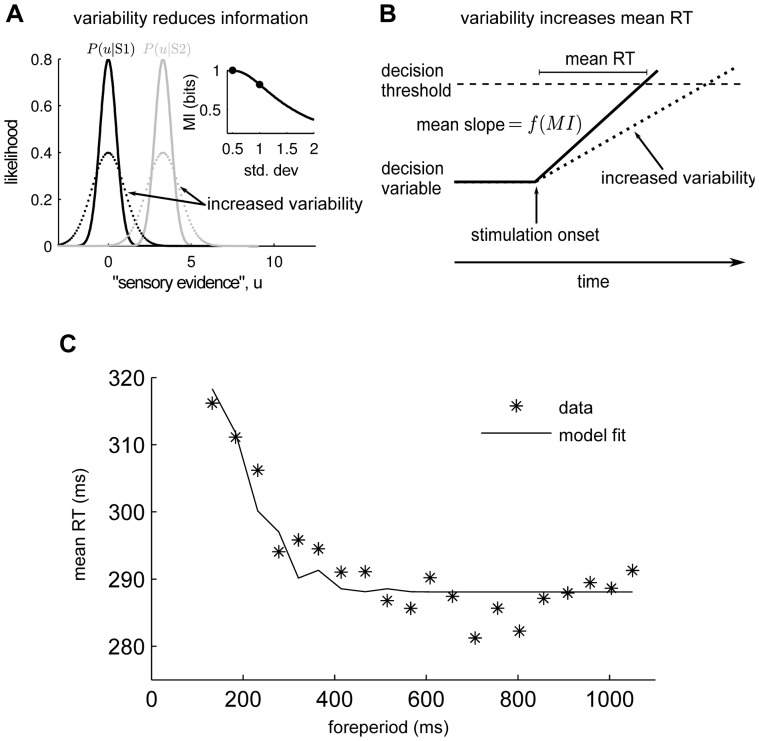
Phenomenological model links variability in sensory responses to response time. (**A**) Example showing that an increase in variability leads to a decrease in mutual information. Two stimuli (S1,S2) are presented with equal probability. This generates ‘sensory evidence’ (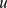) which is used to make the decision (here 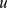 taken as a continuous one-dimensional variable). The variability in the ‘sensory evidence’ is illustrated by plotting the likelihood functions (black for S1, gray for S2). These functions are taken as normal distributions with a standard deviation of 0.5 (solid lines) or 1 (dotted lines). The inset shows how the mutual information depends on the standard deviation of the likelihood functions. (**B**) Illustration of a qualitative model of how mean response time depends on mutual information. At stimulus onset a decision variable starts moving towards a decision threshold (dashed line). The response time is the time between stimulus onset and reaching threshold. The mean rate of rise (slope) of the decision variable is a monotonously increasing function of the mutual information. The different slopes for ‘sensory evidence’ with low variance (solid line) compared to that with higher variance (dotted line) leads to different mean response times. (**C**) Variability at an extrastriate site in monkey T (black dot in in Figure 4B) can account for how mean response times depend on foreperiod duration. Variability of the VEP was converted to mutual information (see Material and Methods) and subsequently used to fit the mean response times.

Information theory is a standard tool used to describe how sensory information is coded in neural activity [34]. In particular, the *mutual information* is often used to quantify the amount of information that neural activity carries about sensory stimuli. Intuitively, the mutual information between a stimulus 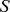 and a response 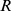 (denoted 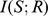) is a measure of the amount of overlap in the response distributions corresponding to different stimuli. If the trial-to-trial variability is conceptualized as noise added to the ‘true’ neural response, the mutual information between stimuli and responses will decrease with increasing variability. Indeed, if we let 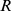 denote the neural response, 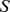 the stimulus, and 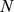 the random trial-to-trial variability (noise), the so-called data processing inequality [35] implies that 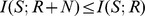. In words, as trial-to-trial variability increases the mutual information between stimuli and responses decreases. The relation between variance and mutual information is schematically illustrated in Figure 6A which shows a simple example with just two possible stimuli and a one-dimensional response distribution.

To connect changes in mutual information to behavior (response time and percent correct responses) we need to consider models of how perceptual decisions are formed. Many such models have been suggested and most postulate a decision variable that changes with time until it reaches a threshold [36]. In such models the behavioral response time is the time between stimulus onset and time of hitting the threshold (see Figure 6B). Such models can often account for the behavioral data, they can be rigorously related to biophysically realistic models of neuronal circuits [37], and moreover, evidence has recently been found for such decision variables in the activity of the cerebral cortex [38], [39]. Two of the most influential models of this type postulate that the mean rate of change of the decision variable is a monotonic function of the mutual information between the stimulus and the ‘sensory response’ [40], [41]. According to these models then, a decrease in the mutual information will lead to a decrease in the mean slope of the decision variable, which will lead to longer mean response times (see Figure 6B). Due to its simplicity, and since the stimuli in our experiment were brief and supra-threshold, we used one of these models, called LATER, e.g. [41],to generate response times from our data.

To fit this model to our data we first converted the measured variability to mutual information. This was done by constructing a simple signal-detection-theory model of our experiment and adjusting the parameters so that the measured VEP variability resulted in approximately the measured percent correct responses (see Material and Methods). This model gave the correspondence between VEP variability and mutual information. Next we fitted a linear function of the estimated mutual information to the inverse of the response time distribution and the resulting fit is shown in Figure 6C.

It is clear that this model can account for most of the variability in the mean response time data (Figure 6C). This supports our interpretation that variability in visual evoked responses interferes with sensory perception and behavioral performance.

## Discussion

Cortical neuronal spiking variability is known to be reduced by stimulus onset [7] and can be further reduced if the stimulus is behaviorally relevant [8]–[10]. The latter effect is characteristically delayed with respect to stimulus onset and has been interpreted as a potential correlate of visual selective attention [8]–[10]. Our results indicate that neural response variability in behaving non-human primates can be controlled *in advance of* stimulus presentation, and the concomitant reduction in mean response times indicate that subjects ‘tune’ their cortical responses to optimize performance. We found that VEP variability reduction was evident in widespread cortical areas but the effect tended to be strongest in visual areas, suggesting that there may be regional specificity in the underlying mechanism. Moreover, our work suggests that reduction in inter-neuronal noise correlations is possible between neuronal populations at distant cortical sites as well as between nearby neurons. A substantial part of this variability and co-variability reduction can be explained by a concomitant reduction in small saccadic eye movements. Indeed, we showed that when human subjects perform the same task the rate of small eye movements decreases as a function of foreperiod duration, and moreover that manual response times are correlated with eye position variability.

Our use of cortical field potentials to quantify neural variability has the advantage of measuring neuronal population activity and hence being sensitive to subtle shifts in neuronal responses across the population. To the extent that the LFP dynamics are a reflection of the membrane potential dynamics of single neurons (e.g. [42]) our findings likely imply a decrease in the spike-count variability of single neurons with increasing foreperiod duration (see Information S1). A reduction in spike-count variability through a reduction in the amplitude of ongoing activity has previously been suggested as a possible mechanism of selective attention [9]. Our results indicate that it could also be used to control stimulus-evoked cortical variability.

We note that pressing the lever to start the trial (which marks the beginning of the foreperiod) is likely to have neural correlates in the cerebral cortex. Since the shortest foreperiod is 100 ms it is possible that neural activity related to lever pressing contributes to the variability of the LFPs that we have described (i.e. on trials with short foreperiods there might be signals related to lever pressing in the data, whereas in trials with longer foreperiods these signals might have ‘died out’). Although we can not completely exclude this explanation given the data we have, there are a number of reasons to believe that neural activity related to lever pressing cannot account for all the findings we report. First, the effects we see are strongest in visual areas, including striate cortex (V1). Pressing a lever, unrelated to visual input, is unlikely to be correlated with a substantial neural response in visual areas. Indeed, in a previous study looking at differences in the average LFPs between GO and NOGO trials in the same data set, we found only minor differences related to lever pressing in early visual areas [32]. Moreover, although variability induced by neural activity related to lever pressing should have led to strong effects in LFPs recorded from somatosensory and motor areas, of four sites located in the vicinity of the central sulcus we found a significant effect of foreperiod duration at only one site (Figure 1). Second, in the experiment where we quantified small eye movements we found that there was a clear effect of foreperiod duration on saccade rates (Figure 5). Since eye movements are known to influence variability in early visual areas we believe that the LFP effects we report are more related to eye movements than to lever pressing. Third, from the literature it is known that foreperiod duration has an effect on mean response times very similar to what we report [27], [43]. In particular, very short foreperiods do not seem to prolong response times compared to trials either without warning cues [43], or with very long foreperiods [27]. That is, it appears that a cue indicating when the stimulus will appear is helpful, and our results can be taken to indicate that this is also the case when the cue is self-initiated (i.e. lever press). Fourth, our interpretation is supported by more recent work in human subjects showing that power in the alpha band of the EEG predicts whether a visual stimulus will be detected or not [44], and is modulated by covert shifts of visual attention [45]. So even if we cannot entirely exclude the possibility that neural activity directly related to lever pressing might be responsible for some of the effects we see (e.g. in frontal regions), it is very unlikely that this is the sole explanation of the very strong effects we found in the visual areas.

We have focused on the variability of the LFP amplitudes. For the visually evoked potential it is possible that the mean activity level also change as a function of foreperiod duration. We found that foreperiod modulations of the average VEP amplitudes did occasionally occur but were much less consistent over stimulus types, recording sites and subjects and are therefore not reported here.

The LFP results we have reported were based on analyzes of preprocessed data (see Material & Methods). If foreperiod duration would systematically influence either the prestimulus mean level or slow (linear) trends in the data it is possible that the preprocessing could bias the results. In Information S1 we therefore apply two alternative measures of variability, measures that can be applied without first preprocessing the data. This analysis showed that the foreperiod-dependent variability reduction was evident also in the ‘raw’ data, demonstrating that the results reported here are not artifacts of the preprocessing.

In our data, VEP variability is drastically reduced during the first 300–400 ms after trial start and reaches a minimum level after some 700–800 ms (Figure 1F). The reduction in mean response time follows a similar time-course (Figure 4A). We have moreover shown that VEP variability depends on variability in ongoing cortical activity, suggesting that control over visual cortical response variability is implemented through control over ongoing cortical activity. Stimulating the nucleus basalis in anesthetized rats leads to reduction of variability in ongoing cortical activity with similar, but slightly slower, time-course [24], suggesting a possible involvement of ascending modulatory systems in our case as well. Indeed, existing evidence suggests that activation of ascending systems improves performance in visual discrimination tasks [46]. An important contribution of the present work is to show that reduced cortical variability does in fact contribute to improvement in behavioral performance.

Visual cortical response variability depends on small fixational eye movements [11], [47], a fact that suggests an alternative, but not mutually exclusive, route to reduced cortical variability, namely reduced rate of fixational eye movements. In fact, it has been shown that the rate of microsaccades can be reduced by a warning cue in attention tasks [48] and the time course of this reduction fits with that of the variability reduction we report. Since fine eye movements were not recorded in the monkey LFP experiments, we investigated the eye movement patterns in this task in two human subjects. The results from this experiment show that the frequency of small saccades decreases as a function of foreperiod duration and that eye movement frequency is correlated with response times on a trial-by-trial level. It thus seems likely that, at least part of, the variability reduction seen in the LFP data is due to a reduction in the propensity to make eye movements. The relation between eye movements and activation of ascending modulatory systems is not well understood but it seems likely that cortical variability reduction may occur both directly due to cortical innervation by ascending systems and indirectly through reduced fixational eye movements.

It is not known how neural response variability could interfere with behavioral performance. We combined the VEP variability in our data with a standard model of perceptual decision making and showed that this could account for how the mean response times depend on foreperiod duration (Figure 6). This model supports the notion that neural variability limits the fidelity of cortical representations of sensory stimuli. Presumably neural variability will prove to have equally detrimental effects on other cortical ‘computations’ and is therefore a critical variable to monitor and control.

## Materials and Methods

We analyzed data from two young adult rhesus macaque monkeys (*Macaca mulatta*) performing a visual GO/NOGO task (subjects T and L). These data are part of a larger data set acquired at the Laboratory for Neuropsychology at the NIMH during 1984–1988. Animal care was in accordance with institutional guidelines at the time. The experimental procedures have been previously described [32], [49] and only a concise description is given here.

### Behavioral task

Subjects initiated a trial by pressing a lever with their preferred hand. At a variable time thereafter a visual stimulus appeared briefly (100 ms) on a screen facing the subject. There were a total of four different stimuli that could be presented and the stimulus-response contingencies were changed within sessions. In a particular stimulus-response contingency, two of the stimuli were associated with the ‘GO’ response and the subjects received a small amount of water if they released the lever within a time window of 500 ms after stimulus onset. The other two stimuli were associated with the ‘NOGO’ response which implied that subjects had to keep the lever pressed. (Correct NOGO trials were not rewarded). The critical variable for the work presented here is the time interval between the (self initiated) lever press and the onset of the visual stimulus. This interval varied randomly between 100 and 1100 ms according to a rectangular distribution over 60 equally sized bins. We will refer to this time interval as the ‘foreperiod’ in analogy to usage in the reaction time literature. Figure 1A shows a schematic illustration of the main events in the task.

### Electrode placement and data acquisition

Each monkey had up to 35 bipolar electrodes chronically implanted transcortically in the hemisphere contralateral to the preferred hand. In each session a subset of 16 the implanted electrodes were connected to Grass P511 amplifiers. The data were band-pass filtered from 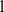 to 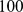 Hz (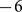 dB at 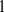 and 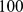 Hz and 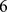 dB per octave falloff) and digitized at 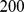 Hz. Data from each electrode were screened by eye and electrodes with large artifacts were excluded from the analysis. This resulted in data simultaneously recorded from 15 and 14 electrodes in subject T and L respectively. Figure 1B illustrates the approximate location of the electrodes used in this study. On each trial, data acquisition started approximately 120 ms before stimulus onset irrespective of foreperiod and lasted for 900 ms. Brain activity during inter-trial intervals were not recorded.

### Data analysis

We analyzed data from 7 and 5 sessions for subject T and L, corresponding to a total of 3847 and 2814 trials respectively. The data sets analyzed are the same as those analyzed in a previous publication [32]. All analyzes were made for each subject separately.

The trials were divided into 20 non-overlapping groups on the basis of foreperiod duration. Trials were further grouped on the basis of stimulus type leading to four groups of trials per foreperiod group (one per stimulus type). To avoid biases in later analyzes we used the same number of trials in all groups. This led to 46 and 34 trials per stimulus type and foreperiod group for subject T and L respectively. In cases where there were more trials for a particular stimulus type and foreperiod group, the adequate number of trials was selected randomly. Since the experiment was balanced with respect to stimulus types and the foreperiod had a rectangular distribution, this restriction on the number of trials per group led only to a ‘loss’ of a small fraction of trials.

#### LFP data

Local field potential data were (semi-automatically) screened for artifacts and outliers. Trials with either were removed. The temporal average during the (100 ms) pre-stimulus interval was subtracted from each trial and linear trends were removed by fitting a line to the mean corrected LFP data from each trial (900 ms) and subsequently subtracting this line from the data. Sixty Hz power-line contaminations were removed using a notch filter.

For the analysis of across-trial variance we first calculated the sample variance for each time point for each group of trials and each of the stimulus types separately. This gave for each of the 20 foreperiod groups four time series of variance values (one for each stimulus type). For the analysis underlying Figure 1, C–H we computed the average of these variances in a 50 ms time window covering the early part of the stimulus evoked response, i.e. the VEP (60–110 ms for monkey T and 75–125 ms for monkey L). Next we computed the Spearman rank correlation between these averages and mean foreperiod duration for the same groups of trials. This step was done for each stimulus type separately. Finally we computed the average of these rank correlations over stimulus types (results presented in Figure 1G). To assess the statistical significance of these correlations we repeated the same steps 10000 times on surrogate data for which we randomly permuted the foreperiod durations between the 20 groups. We then compared the empirical value of the average correlation coefficient to the appropriate values in the tails of the permutation distribution. We corrected for multiple comparisons (number of recording sites) by dividing the desired 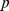-value (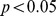, two-sided test) by the corresponding number of sites (29).

For the analysis of noise correlations (Figure 1F) we followed the same approach but with the variance within recording sites replaced by the rank correlation between sites. We restricted the analysis to those site pairs having a significant modulation of the variance. Statistical significance was again assessed by a permutation procedure and the p-values were corrected for the number of pairs (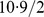 for monkey T and 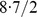 for monkey L).

The analysis of the prestimulus across-trials variability (Figure 2) was performed analogously but using LFP data from a 100 ms time-window extending from 90 ms before stimulus onset to 10 ms after. The spectral analysis shown in Figure 2 was performed on the same data. The amplitude spectra were estimated from single trials and subsequently averaged. Due to the short duration of the data window (100 ms) we could not resolve frequencies below 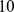 Hz and the contribution of slower frequencies to the estimated amplitudes at 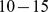 Hz may have been substantial.

For analysis of the dependence between variance in the prestimulus period and the post stimulus period (Figure 3) single trial estimates of variability were required. For the data from the prestimulus time window we used the sample variance computed over time points. To have a single trial estimate of the variability of the stimulus evoked response we first computed the average response for each group of trials. Then for each trial in a particular group we computed the squared deviation from this average for each time point. We then computed the average over the time window of interest (same as above) and took the square root. This gave the mean deviation from the average evoked response and was hence an appropriate measure of single trial variability. The correlation between prestimulus and evoked variability was computed after the effect of foreperiod duration was partialed out (partial correlation analysis). The statistical significance was again assessed by a procedure where the trial labels were randomly permuted. The p-values were corrected for the number of comparisons made.

The correlation between variability of the evoked response and response times (Figure 3B) was done analogously.

The test of the statistical significance of the response time modulation (Figure 4A) was made by comparing the rank correlation between mean response time and mean foreperiod with that obtained by randomly permuting the foreperiod groups.

### Eye movement data

The same task performed by the monkey subjects was implemented for humans in Matlab using the Psychophysics Toolbox [50], [51] with the space key of a standard keyboard serving as response lever. Since we were primarily interested in how response times were modulated by eye movements and foreperiod durations, the timing precision offered by a standard keyboard was deemed sufficient.

Eye movements were recorded with the Eyelink 1000 (SR Research, Canada), an up-to-date camera-based eye tracker, used in a monocular tower-mount configuration. Horizontal and vertical positions of the right eye were recorded at a sampling rate of 1KHz and with a spatial accuracy better than 0.1 degrees of visual angle. The eyetracker was interfaced with the EyelinkToolbox [52], provided with the PsychToolbox3 [50], [51], under Matlab. Saccades were detected by thresholding the eye-velocity traces at 20 degrees per second. Eye velocity was estimated by computing the numerical derivative of the smoothed eye position traces using a second order method. The number of trials in the two subjects were 600 and 800 respectively. To test if eye movements were influenced by foreperiod duration we used the standard deviation of the position trace (within a 200 ms time window) as a proxy for eye movements. This measure correlated highly with saccade rate, but since the latter is a quantity computed over trials we used the former to enable a trial-by-trial analysis.

### Model of the relation between VEP variability and mean response times

To account for one of our main findings, that VEP variability is correlated with response times, we constructed a simple model that allowed us to use measured VEP variability to predict response times. We used a standard model (LATER) of how sensory stimuli controls response times [41], [53]. This model is not formulated in terms of neural activity (or the variance of neural activity) and in order to apply the model in our setting we first needed to connect VEP variability to a variable in the model. The key variable that determines the mean response times in the LATER model is the slope (rate of rise) of the decision variable. This slope is connected to the mutual information between stimuli and responses and we therefore devised a way to connect VEP variability to mutual information.

To connect variability to mutual information we first designed a signal detection theory model for our experiment where it is assumed that each stimulus gives rise to a 2-dimensional decision variable described by a symmetric Gaussian distribution. We then modulated the variances of these 2D Gaussians using the variability measured in the data. The remaining parameter in the model, the location of the four distributions, was changed until the model could reproduce the performance (in terms of percent correct ‘responses’) of the animals. For a given level of variance of the Gaussian distributions we calculated the mutual information using Monte Carlo methods.

For the fit shown in Figure 6C the four Gaussian profiles were centered on the corners of a square with edges having a length of 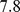 (a.u.). The distributions had a nominal variance of 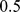. The variance of the stimulus evoked response from an extrastriate channel (black dot in Figure 4B) was normalized to have maximum of 1 and subsequently used to scale the four distributions. We then found the best (in least squares sense) linear relation between the estimated values of mutual information and mean response time and this is what is shown in Figure 6C.

The signal detection model we have used to connect VEP variability to mutual information is somewhat arbitrary in the sense that other models would have given a similar relationship between the two variables. This model is not intended as a faithful description of how the stimuli are represented in the brain. Rather it is a convenient way to convert variability to mutual information. It should be emphasized that a fit equally good to that shown in Figure 6C can be obtained by modeling response time directly as a function of VEP variability.

## Supporting Information

Information S1 **Supporting figures and text.**
(PDF)

## References

[1] Werner G, Mountcastle VB (1963) Variability of central neural activity in a sensory system, and its implications for central reection of sensory events. J Neurophys 26: 958–977. 10.1152/jn.1963.26.6.958 14084169

[2] Whitsel BL, Schreiner RC, Essick GK (1977) Analysis of variability in somatosensory cortical neuron discharge. J Neurophys 40: 589–607. 10.1152/jn.1977.40.3.589 406367

[3] Tolhurst DJ, Movshon JA, Dean AF (1983) The statistical reliability of signals in single neurons in cat and monkey visual-cortex. Vision Res 23: 775–785. 6623937 10.1016/0042-6989(83)90200-6

[4] Vogels R, Spileers W, Orban GA (1989) The response variability of striate cortical-neurons in the behaving monkey. Exp Brain Res 77: 432–436. 2792290 10.1007/BF00275002

[5] Gawne TJ, Richmond BJ (1993) How independent are the messages carried by adjacent inferior temporal cortical-neurons. J Neurosci 13: 2758–2771. 8331371 10.1523/JNEUROSCI.13-07-02758.1993 PMC6576676

[6] Zohary E, Shadlen MN, Newsome WT (1994) Correlated neuronal discharge rate and its implications for psychophysical performance. Nature 370: 140–143. 8022482 10.1038/370140a0

[7] Churchland MM, Yu BM, Cunningham JP, Sugrue LP, Cohen MR, et al (2010) Stimulus onset quenches neural variability: a widespread cortical phenomenon. Nat Neurosci 13: 369–378. 20173745 10.1038/nn.2501 PMC2828350

[8] Mitchell JF, Sundberg KA, Reynolds JH (2007) Differential attention-dependent response modulation across cell classes in macaque visual area v4. Neuron 55: 131–141. 17610822 10.1016/j.neuron.2007.06.018

[9] Mitchell JF, Sundberg KA, Reynolds JH (2009) Spatial attention decorrelates intrinsic activity uctuations in macaque area v4. Neuron 63: 879–888. 19778515 10.1016/j.neuron.2009.09.013 PMC2765230

[10] Cohen MR, Maunsell JHR (2009) Attention improves performance primarily by reducing interneuronal correlations. Nat Neurosci 12: 1594–1600. 19915566 10.1038/nn.2439 PMC2820564

[11] Gur M, Beylin A, Snodderly DM (1997) Response variability of neurons in primary visual cortex (V1) of alert monkeys. J Neurosci 17: 2914–2920. 9092612 10.1523/JNEUROSCI.17-08-02914.1997 PMC6573112

[12] Wurtz RH (1969) Response of striate cortex neurons to stimuli during rapid eye movements in the monkey. J Neurophys 32: 975–986. 10.1152/jn.1969.32.6.975 4981518

[13] Leopold D, Logothetis N (1998) Microsaccades differentially modulate neural activity in the striate and extrastriate visual cortex. Exp Brain Res 123: 341–345. 9860273 10.1007/s002210050577

[14] Martinez-Conde S, Macknik S, Hubel D (2000) Microsaccadic eye movements and firing of single cells in the striate cortex of macaque monkeys. Nat Neurosci 3: 251–258. 10700257 10.1038/72961

[15] Rajkai C, Lakatos P, Chen CM, Pincze Z, Karmos G, et al (2008) Transient cortical excitation at the onset of visual fixation. Cereb Cortex 18: 200–209. 17494059 10.1093/cercor/bhm046

[16] Bosman CA, Womelsdorf T, Desimone R, Fries P (2009) A Microsaccadic Rhythm Modulates Gamma-Band Synchronization and Behavior. J Neurosci 29: 9471–9480. 19641110 10.1523/JNEUROSCI.1193-09.2009 PMC6666524

[17] Rolfs M, Kliegl R, Engbert R (2008) Toward a model of microsaccade generation: The case of microsaccadic inhibition. J Vis 8: 5: 1–23. 10.1167/8.11.5 18831599

[18] Hafed ZM, Lovejoy LP, Krauzlis RJ (2011) Modulation of Microsaccades in Monkey during a Covert Visual Attention Task. J Neurosci 31: 15219–15230. 22031868 10.1523/JNEUROSCI.3106-11.2011 PMC3229866

[19] Arieli A, Sterkin A, Grinvald A, Aertsen A (1996) Dynamics of ongoing activity: Explanation of the large variability in evoked cortical responses. Science 273: 1868–1871. 8791593 10.1126/science.273.5283.1868

[20] Steriade M (2001) Impact of network activities on neuronal properties in corticothalamic systems. J Neurophysiol 86: 1–39. 11431485 10.1152/jn.2001.86.1.1

[21] Ferezou I, Haiss F, Gentet LJ, Aronoff R, Weber B, et al (2007) Spatiotemporal dynamics of cortical sensorimotor integration in behaving mice. Neuron 56: 907–923. 18054865 10.1016/j.neuron.2007.10.007

[22] Moruzzi G, Magoun HW (1949) Brain stem reticular formation and activation of the EEG. Electroencephalogr Clin Neurophysiol 1: 455–473. 18421835

[23] Jasper HH (1954) The concept of attention and the electroencephalographic alpha rythm. In: Jasper HH, editor, Brain Mechanisms and Conciousness, Oxford: Blackwell.

[24] Goard M, Dan Y (2009) Basal forebrain activation enhances cortical coding of natural scenes. Nat Neurosci 12: 1444–1449. 19801988 10.1038/nn.2402 PMC3576925

[25] Lansing RW, Schwartz E, Lindsley DB (1959) Reaction-time and EEG activation under alerted and nonalerted conditions. J Exp Psychol 58: 1–7. 13664877 10.1037/h0041016

[26] Bertelson P (1967) Time course of preparation. Q J Exp Psychol 19: 272–279. 6074169 10.1080/14640746708400102

[27] Green DM, Smith AF, Vongierke SM (1983) Choice reaction-time with a random foreperiod. Percept Psychophys 34: 195–208. 6646960 10.3758/bf03202946

[28] Purpura DP (1959) Nature of electrocortical potentials and synaptic organizations in cerebral and cerebellar cortex. In: Pfeiffier CC, Smythies JR, editors, International Review of Neurobiology, New York: Academic Press, volume 1. pp. 47–163. 10.1016/s0074-7742(08)60314-1 14435355

[29] Mitzdorf U (1985) Current source-density method and application in cat cerebral cortex: investigation of evoked potentials and EEG phenomena. Physiol Rev 65: 37–100. 3880898 10.1152/physrev.1985.65.1.37

[30] Kraut MA, Arezzo JC, Vaughan HG Jr (1985) Intracortical generators of the ash VEP in monkeys. Electroencephalogr Clin Neurophysiol 62: 300–12. 2408876 10.1016/0168-5597(85)90007-3

[31] Schroeder CE, Tenke CE, Givre SJ, Arezzo JC, Vaughan HG Jr (1991) Striate cortical contribution to the surface-recorded pattern-reversal in the alert monkey. Vision Res 31: 1143–57. 1891808 10.1016/0042-6989(91)90040-c

[32] Ledberg A, Bressler SL, Ding MZ, Coppola R, Nakamura R (2007) Large-scale visuomotor integration in the cerebral cortex. Cereb Cortex 17: 44–62. 16452643 10.1093/cercor/bhj123

[33] Averbeck BB, Latham PE, Pouget A (2006) Neural correlations, population coding and computa- tion. Nat Rev Neurosci 7: 358–366. 16760916 10.1038/nrn1888

[34] Rieke F, Warland D, de Reuter van Stevenick R, Bialek W (1997) Spikes: Exploring the neural code. Cambridge: MIT-Press.

[35] Cover TM, Thomas JA (1991) Elements of Information Theory. New York: Wiley.

[36] Luce RD (1986) Response times. New York: Oxford University Press, first edition.

[37] Roxin A, Ledberg A (2008) Neurobiological models of two-choice decision making can be reduced to a one-dimensional nonlinear diffusion equation. PLoS Comput Biol 4: e1000046. 18369436 10.1371/journal.pcbi.1000046 PMC2268007

[38] Schall JD (2001) The neural basis of deciding, choosing and acting. Nat Rev Neurosci 2: 33–42. 11253357 10.1038/35049054

[39] Gold JI, Shadlen MN (2007) The neural basis of decision making. Annu Rev Neurosci 30: 535–574. 17600525 10.1146/annurev.neuro.29.051605.113038

[40] Ratcliff R, McKoon G (2008) The diffusion decision model: Theory and data for two-choice decision tasks. Neural Comput 20: 873–922. 18085991 10.1162/neco.2008.12-06-420 PMC2474742

[41] Carpenter RHS, Reddi BAJ, Anderson AJ (2009) A simple two-stage model predicts response time distributions. J Physiol 587: 4051–4062. 19564395 10.1113/jphysiol.2009.173955 PMC2756437

[42] Steriade M (1997) Synchronized activities of coupled oscillators in the cerebral cortex and thalamus at different levels of vigilance. Cereb Cortex 7: 583–604. 9276182 10.1093/cercor/7.6.583

[43] Bertelson P, Tisseyre F (1969) Time-course of preparation - confirmatory results with visual and auditory warning signals. Acta Psychologica 30: 145–154.

[44] Ergenoglu T, Demiralp T, Bayraktaroglu Z, Ergen M, Beydagi H, et al (2004) Alpha rhythm of the EEG modulates visual detection performance in humans. Cognit Brain Res 20: 376–383. 10.1016/j.cogbrainres.2004.03.009 15268915

[45] Thut G, Nietzel A, Brandt SA, Pascual-Leone A (2006) *α*-band electroencephalographic activity over occipital cortex indexes visuospatial attention bias and predicts visual target detection. J Neurosci 26: 9494–9502. 16971533 10.1523/JNEUROSCI.0875-06.2006 PMC6674607

[46] Fuster JM, Uyeda AA (1962) Facilitation of tachistoscopic performance by stimulation of midbrain tegmental points in monkey. Exp Neurol 6: 384–406. 13945940 10.1016/0014-4886(62)90020-1

[47] Gur M, Snodderly DM (2006) High response reliability of neurons in primary visual cortex (v1) of alert, trained monkeys. Cereb Cortex 16: 888–895. 16151177 10.1093/cercor/bhj032

[48] Engbert R, Kliegl R (2003) Microsaccades uncover the orientation of covert attention. Vision Res 43: 1035–1045. 12676246 10.1016/s0042-6989(03)00084-1

[49] Bressler SL, Nakamura R (1993) Interarea synchronization in macaque neocortex during a visual pattern discrimination task. In: Eeckman FH, Bower JM, editors, Computation and Neural Systems, Boston: Kluwer. pp. 515–22.

[50] Brainard DH (1997) The psychophysics toolbox. Spat Vis 10: 433–436. 9176952

[51] Pelli DG (1997) The videotoolbox software for visual psychophysics: Transforming numbers into movies. Spat Vis 10: 437–442. 9176953

[52] Cornelissen F, Peters E, Palmer J (2002) The eyelink toolbox: Eye tracking with matlab and the psychophysics toolbox. Behav Res Meth Ins C 34: 613–617. 10.3758/bf03195489 12564564

[53] Reddi BAJ, Carpenter RHS (2000) The inuence of urgency on decision time. Nat Neurosci 3: 827–830. 10903577 10.1038/77739

