# Supplementary material for: Reduced Variability of Ongoing and Evoked Cortical Activity Leads to Improved Behavioral Performance
Source: PLoS One. 2012 Aug 24;7(8):e43166. doi: 10.1371/journal.pone.0043166 (PMC3427304; doi:10.1371/journal.pone.0043166)
Supplement: Information S1 — Supporting figures and text. (PDF) [file pone.0043166.s001.pdf]

Supporting Information for

Reduced variability of ongoing and evoked cortical activity leads to improved behavioral performance

Anders Ledberg, Anna Montagnini, Richard Coppola, and Steven Bressler

June 3, 2012

Contents

1 Modulations of prestimulus and VEP variability are visible also in raw data 2

1.1 Results 2

1.1.1 VEP variability 2

1.1.2 Prestimulus LFP variability 2

2 Increased variability in the local field potential increases spiking variability 5

List of Figures

S1 VEP variability modulation is not dependent on preprocessing . 3

S2 Prestimulus variability modulation is not dependent on preprocessing 4

S3 Low frequency variability of the membrane potential increases single neuron response irregularity 6

1 Modulations of prestimulus and VEP variability are visible also in raw data

All the analyzes made in the main paper were done after the data from each trial was preprocessed to have zero mean in the first 100 ms and no linear trends (computed over the complete 900 ms data for each trial and recording site). If foreperiod duration influences the mean level of the signal or the slow (linear) drift then this preprocessing could introduce a bias in the results. To show that this was not the case we here device two procedures through which variability can be assessed in data not preprocessed (called 'raw data' in the following). To estimate the variability in the prestimulus window we can use the sample variance computed over time instead of over trials. That is, for each trial we take the first 20 samples and compute the sample variance of these. To the extent that the LFPs in the prestimulus window can be approximated by a stationary stochastic process this 'across-time' estimate will be highly correlated with the across-trials variance estimate we use the main paper. To assess VEP variability in the raw data we proceeded as follows. For a given trial and recording site we look up the largest and smallest value of the LFPs in the 50 ms window that covers the early part of the VEP (same windows as in the main paper). The difference between these two numbers then serves as a rough estimate of the VEP amplitude. This gives us one VEP amplitude per trial and site and we subsequently compute the sample variance of these amplitudes in groups of trials sorted with respect to foreperiod duration.

1.1 Results

1.1.1 VEP variability

Of all the 17 sites where the VEP variance was deemed to decrease with respect to foreperiod duration (i.e. all 'significant' sites show in in Figure 1G in the main paper) all but two (both in monkey L) had decreasing variances also when estimated directly on the raw data. This shows that the decrease in VEP variance reported is unlikely to reflect an artifact of the data preprocessing used. Figure S1 exemplifies this and shows that VEP variance estimated from the raw data shows similar modulation with respect to foreperiod duration as does VEP variance estimates derived from the preprocessed data (i.e. the results reported in the main text).

1.1.2 Prestimulus LFP variability

The (across-time) estimates of prestimulus variability obtained from the raw data were very similar to the estimates computed over trials in the processed data. Here all sites with a decreasing variance as a function of foreperiod duration also showed decreasing variance when this was estimated from the raw data. Figure S2 shows examples of this from three different recording sites. Note that the numerical values obtained are very similar to the ones obtained from the preprocessed data, showing that the preprocessing has minimal effect on the variability of the prestimulus epoch.

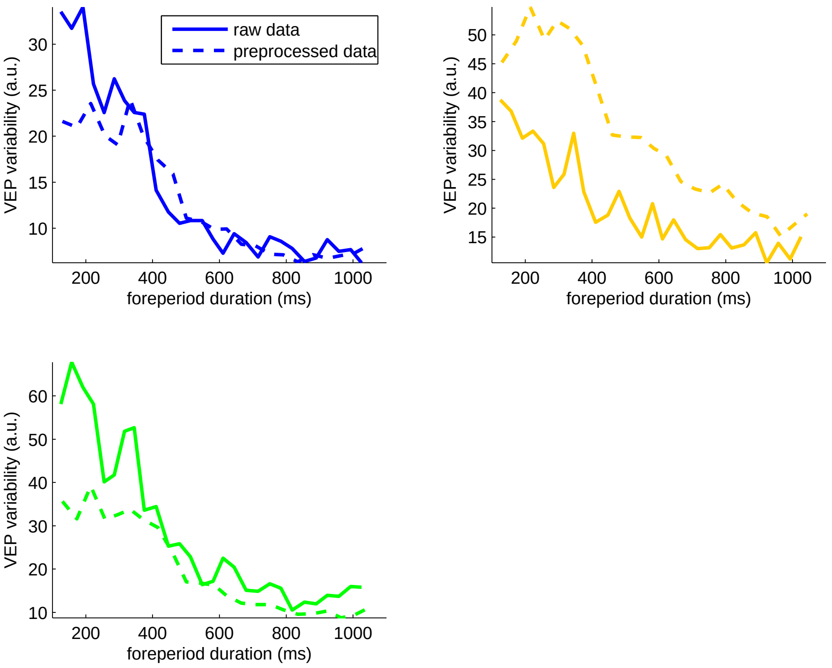

Figure S1: VEP variability modulation is not dependent on preprocessing of the data. The three panels show two different estimates of VEP variability for the three sites indicated in Figure 1F in the main text (same color code as in that Figure). The 'raw data' estimates were computed as indicated in the supplementary information (this document) and the 'preprocessed data' estimates are identical to what is shown in the main text (reproduced here for comparison). The raw data estimates have been scaled by 1/2 to make comparisons easier.

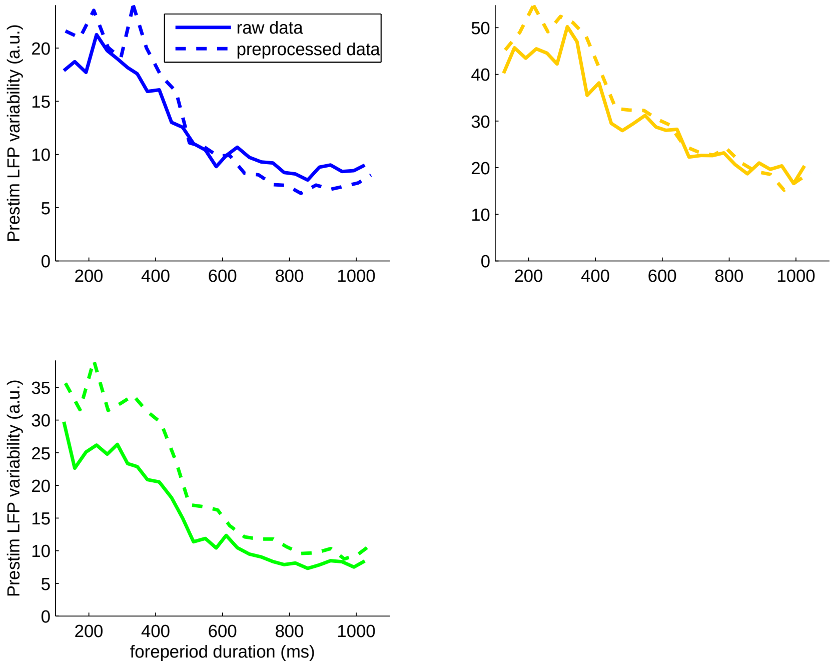

Figure S2: Prestimulus variability modulation is not dependent on preprocessing of the data. The three panels show two different estimates of the prestimulus variability for the three sites indicated in Figure 1F in the main text (same color code as in that Figure). The 'raw data' estimates were computed as indicated in the supplementary information (this document) and the 'preprocessed data' estimates are identical to what is shown in the main text, Figure 2A (reproduced here for comparison).

2 Increased variability in the local field potential increases spiking variability

Here we investigate the possible effects of changes in the variability of the local field potential on single neuron spiking variability. Cortical field potentials are believed to reflect mainly local synaptic activity [Purpura, 1959, Mitzdorf, 1985]. From experiments using simultaneous intracellular and field potential recordings it is known that the membrane potentials of single neurons are often highly correlated with the local field (e.g. Steriade [1997], Poulet and Petersen [2008]). It is therefore reasonable to assume that changes in the properties of field potentials will be reflected in the membrane potentials of single cells. Motivated by this viewpoint, we employed a biophysically derived model of a cortical pyramidal cell from the literature [Destexhe et al., 2001]. Starting with the same parameters as used in Destexhe et al. [2001] we studied the effects of adding more variability to the membrane potential. Spectral analysis of prestimulus LFP data indicated that the variability changes due to foreperiod duration were mainly due to activity at low frequencies (see Figure 2 in the main text). At most sites in the 'visual areas' the change was restricted to frequencies below 20 Hz and the most pronounced changes occurred at frequencies below 12 Hz. We therefore thought to determine the effects in the model neuron of spiking regularity on the low frequency content of intracellular variability (see Fig. S3). In particular, we used excitatory and inhibitory conductances described by an autoregressive model of order two and used parameters to match the spectra of the model neuron membrane potential to the spectra of the LFP data (compare the spectras in Figure 2 in the main text with those in Figure S3C). It is important to realize that the injected noisy conductances had zero mean, and only the amplitude of the noise term (innovation term) was changed. Consequently, in all simulations the mean input to the cell was the same. We then studied how the regularity of the spikes evoked by a steady injected current depended on the variability of the conductances. To measure spiking variability we used the sample variance of the spike count (over trials) divided by the mean of the spike count in a time window of 100 ms (this measure is sometimes called the Fano factor). There was indeed a strong effect on the variability on the spiking regularity (Fig. S3D)). This can be intuitively understood in the following way. For trials simulated with a large noise amplitude the membrane potential might be relatively hyperpolarized for a substantial amount of time (due to the slow fluctuations of the noise) and hence the cell fires fewer spikes than on average. On other trials the membrane potential might be relatively depolarized and the cell fires more spikes than on average. Thus increasing the amplitude of fluctuations that are of the same timescale as the analysis window (100 ms) inevitably increases the trial-to-trial spiking variability for certain values of the 'sensory' input current.

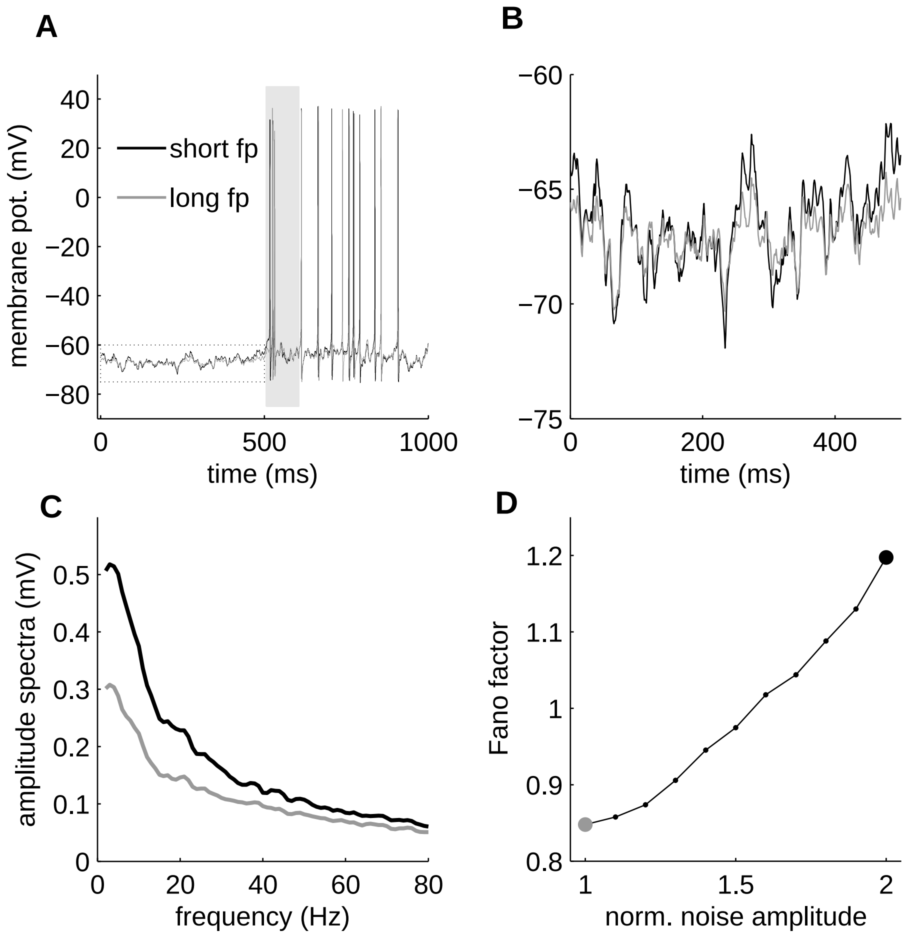

Figure S3: Low frequency variability of the membrane potential increases response irregularity. (**A**) Membrane potential of model neuron for two values of background variability. In black are data corresponding to a short foreperiod (high variability of the membrane potential). In gray are data corresponding to a long foreperiod (lower variability of the membrane potential). At 500 ms the cell is receiving an additional input consisting in a Poisson spike train with a rate of 500 Hz. (**B**) Magnification of the 'prestimulus' part of **A** (i.e. from 0 to 500 ms). (**C**) Amplitude spectra of the background state where the cell is not spiking. Trials with a 'short foreperiod' (i.e. with large membrane potential variability) in black and those with a long foreperiod in gray. Spectras were estimated in non-overlapping windows of 500 ms from a simulation without additional stimulation (to prevent the cell from spiking). (**D**) Fano factor as a function of noise intensity. In each trial the number of spikes in a 100 ms window starting at 'stimulus onset' was used to compute the Fano factor (variance of spike count divided by mean of spike count, computed over 1000 'trials'). Changing the window length or moving it within the 'activated' epoch did not change the results qualitatively.

References

S. L. Bressler and R. Nakamura. Interarea synchronization in macaque neocortex during a visual pattern discrimination task. In Frank H. Eeckman and James M. Bower, editors, *Computation and Neural Systems*, pages 515–22. Kluwer, Boston, 1993.

A. Destexhe, M. Rudolph, J. M. Fellous, and T. J. Sejnowski. Fluctuating synaptic conductances recreate in vivo-like activity in neocortical neurons. *Neuroscience*, 107:13–24, 2001.

A. Ledberg, S. L. Bressler, M. Z. Ding, R. Coppola, and R. Nakamura. Large-scale visuomotor integration in the cerebral cortex. *Cereb. Cortex*, 17:44–62, 2007.

U. Mitzdorf. Current source-density method and application in cat cerebral cortex: investigation of evoked potentials and EEG phenomena. *Physiol Rev*, 65(1):37–100, Jan 1985.

J. F. A. Poulet and C. C. H. Petersen. Internal brain state regulates membrane potential synchrony in barrel cortex of behaving mice. *Nature*, 454:881–U36, 2008.

Dominick P. Purpura. Nature of electrocortical potentials and synaptic organizations in cerebral and cerebellar cortex. In Carl C. Pfeiffer and John R. Smythies, editors, *International Review of Neurobiology*, volume 1, pages 47–163. Academic Press, New York, 1959.

M. Steriade. Synchronized activities of coupled oscillators in the cerebral cortex and thalamus at different levels of vigilance. *Cerebral Cortex*, 7:583–604, 1997.
